# Genome-wide analysis reveals the contributors to fast molecular evolution of the Chinese hook snout carp (*Opsariichthys bidens*)

**DOI:** 10.1016/j.csbj.2024.05.048

**Published:** 2024-05-31

**Authors:** Fengbo Li, Wei Wang, Haihua Cheng, Ming Li

**Affiliations:** a Zhejiang Institute of Freshwater Fisheries, 999 Hangchangqiao South Road, Huzhou 313001, China; b State Key Laboratory of Integrated Management of Pest Insects and Rodents, Institute of Zoology, Chinese Academy of Sciences, 1 Beichen West Road, Beijing 100101, China; c Jinhua Fisheries Technology Extension Center, 828 Shuanglong South Street, Jinhua 321013, China

**Keywords:** Rate heterogeneity, Accelerated evolution, Life-history trait, Adaptation, Cypriniformes

## Abstract

Variations in molecular evolutionary rate have been widely investigated among lineages and genes. However, it remains an open question whether fast rate of molecular evolution is driven by natural selection or random drift, and how the fast rate is linked to metabolic rate. Additionally, previous studies on fast molecular evolution have been largely restricted to concatenated matrix of genes or a few specifically selected genes, but less is known for individual genes at the genome-wide level. Here we addressed these questions using more than 5000 single-copy orthologous (SCO) genes through comparative genomic and phylogenetic analyses among fishes, with a special focus on a newly-sequenced clupeocephalan fish the Chinese hook snout carp *Opsariichthys bidens*. We showed *O. bidens* displays significantly higher mean substitution rate and more fast-evolving SCO genes (2172 genes) than most fishes studied here. The rapidly evolving genes are enriched in highly conserved and very basic functions such as translation and ribosome that are critical for biological fitness. We further revealed that ∼25 % of these fast-evolving genes exhibit a constant increase of substitution rate from the common ancestor down to the present, suggesting a neglected but important contribution from ancestral states. Model fitting showed that ∼85 % of fast-evolving genes exclusive to *O. bidens* and related species follow the adaptive evolutionary model rather than random-drift model, and 7.6 % of fast-evolving genes identified in *O. bidens* have experienced positive selection, both indicating the reflection of adaptive selection. Finally, metabolic rate was observed to be linked with substitution rate in a gene-specific manner. Overall, our findings reveal fast molecular evolution of SCO genes at genome-wide level in *O. bidens*, and uncover the evolutionary and ecological contributors to it.

## Highlights

• We sequenced the chromosome-level genome of the Chinese hook snout carp *Opsariichthys bidens*.
• *O. bidens* displays higher substitution rate and more fast-evolving genes than most of other fish species studied here.
• The ancestral state contributed to the fast molecular evolution, and the underlying molecular mechanism was suggested.
• Adaptive selection rather than random drift acts as another important contributor to the fast molecular evolution.
• Effect of metabolic rate on substitution rate is gene-specific, shedding light on previous contradictory observations.

## Graphical Abstract

**Figure:**
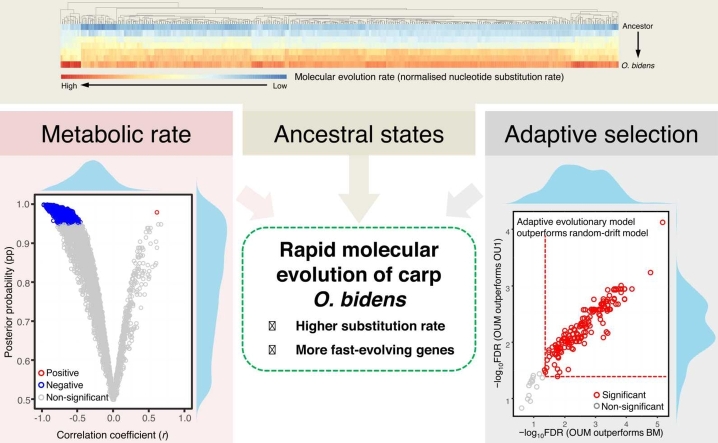

## 1 Introduction

Rate of molecular evolution (mainly referring to the nucleotide substitution rate in this study) is widely known to vary greatly across not only lineages but also genes [6], [8], [91]. Addressing how and why the rate variations occur is very important for understanding fundamental questions in evolutionary biology, e.g. the link between molecular evolution and phenotypic/morphological evolution, the relationship between gene tree and species tree, and contradictions between phylogenomic analyses [72], [75], [8]. While both fast and slow rate may be interesting, lineages/genes at the fast end (i.e. possessing a fast substitution rate) are frequently utilized for detecting evolutionary forces [15], [2], [60], [91] and inferring phylogenetic relationships of animals (e.g. the mitochondrial genes; [38]). In the past decades, comparative genomic approach has largely facilitated the investigation of rapid molecular evolution in various animal groups. For instance, in mammals, orthologous DNA sequences of cercopithecoids experience substitution rate that are much faster than those of hominoids [77]; birds in the order Passeriformes exhibit higher substitution rate than other avian species [102]; in insects, the low-elevation bumblebees have undergone faster genome-wide evolution than the high-elevation ones [42]. However, previous studies mainly focused on concatenated matrix of orthologous genes (sequences), and it remains less clear how the fast rate of molecular evolution is reflected among individual genes at the genome-wide level.

A crucial question following the detection of fast rate is why it happens. Indeed, elucidating the causes of variations in molecular evolution rate has been the subject of substantial studies in evolutionary biology and ecology. Regarding the underlying evolutionary mechanism, both adaptive selection and non-adaptive random drift have been suggested to drive the rapid molecular evolution. Such evolutionary assumptions are frequently used to address the fast evolution of several specifically selected genes (e.g. [26], [87], [104], [30], [45], [91], [66]). For example, reproductive genes display an elevated rate of molecular evolution, which is hypothesized to correlate with either sexual selection or drift due to sex-specific gene expression [17], [83], [91]. To differentiate the two evolutionary mechanisms (adaptive and non-adaptive evolution), an important strategy for traits associated with life history is to conduct phylogenetic comparative analysis. Namely, it is necessary to test whether the trait data follows the adaptive evolutionary model (e.g. Ornstein-Uhlenbeck model) or alternative non-adaptive model (e.g. Brownian motion model) [4]. Although substitution rate can be considered as the trait linked with life-history variation [8], it has not been used to fit these models for genes at the genome-wide level, leading to difficulties in evaluating the contributions of adaptive and non-adaptive processes to fast substitution rate.

Many ecological or life-history traits are also suggested to be responsible for the fast substitution rate, e.g. metabolic rate, fecundity, longevity, generation time, body mass [2], [50], [70], [71], [74], [89], [9]. In particular, the ‘metabolic rate hypothesis’ suggests a positive effect of metabolic rate on substitution rate: lineages with higher metabolic rate tend to produce more oxygen radical that results in more DNA damage and thus show increased substitution rate [49], [89]. This hypothesis was firstly supposed in cartilaginous fishes decades ago [48]. However, subsequent studies have obtained quite complex results, that is, absence of and even negative metabolic rate effect were observed in distinct animal groups [36], [63], [71], [74], [9]. These controversial observations could be due to that metabolic rate may influence the substitution rate in a gene-specific manner [36], [74], namely, different genes respond to metabolic rate in different patterns. However, this hypothesis remains untested in a global framework, i.e. throughout the genome. Investigation of this question will allow for unbiased assessment of how the genome-scale individual genes are influenced by metabolic rate.

To reveal how and why fast molecular evolution happens at the genome-wide level, here we focused on single-copy orthologous (SCO) genes from 12 clupeocephalan fish species where the genome resources and resting metabolic rate (RMR) data are available. Clupeocephala is composed of cohorts Otomorpha (synonym of Otocephala) and Euteleostei (synonym of Euteleosteomorpha) [18], [7]. Based on concatenated matrix of orthologous non-coding sequences, a previous study has reported variations in substitution rate among four clupeocephalan species [39]. It’s still unknown whether such variations can be approved in coding sequences for genome-scale genes in this lineage. Within Clupeocephala, the Chinese hook snout carp *Opsariichthys bidens* (cohort Otomorpha: order Cypriniformes) is an emerging economic fish species in China in recent years. Carp *O. bidens* is known for its ability to grow fast and adapt well to both the field and farming environment, making it an ideal species to investigate adaptation and fast evolution [101], [106], [14], [46]. Currently, genomic studies on *O. bidens* have largely focused on chromosome evolution and adaptive radiation [22], [43], [95]. However, little is known about the molecular evolutionary rate of *O. bidens* and the underlying determinants. In the present study, by newly generating a chromosome-level genome assembly for *O. bidens*, we addressed two questions using comparative genomic and phylogenetic analyses. (I) How does the fast rate of nucleotide substitution happen to protein-coding genes in *O. bidens* at the genome-wide level? (II) Why does the fast evolution happen at the molecular level: is it suggestive of non-adaptive or adaptive evolution, and affected by metabolic rate in a gene-specific manner?

## 2 Materials and methods

### 2.1 Genome sequencing and annotation of the Chinese hook snout carp *O. bidens*

#### 2.1.1 Carp materials and genome sequencing

We generated the whole genome assembly for a female individual of *O*. *bidens*, which was collected from a fishery in Zhejiang Province in China. Carp sample collection and handling procedures followed the current laws of China on the Protection of Wildlife. Muscle tissue of the fish was dissected and subjected to DNA extraction (Qiagen). Genomic DNA quality was checked using NanoDrop-2000 spectrophotometer (Thermo Fisher Scientific), Qubit fluorometer (Thermo Fisher Scientific), and 0.75 % agarose gel electrophoresis. Short- and long-read sequencing were then conducted using Illumina HiSeq (library insert size 350 bp, 2 × 150 bp reads) for genome surveying and base correction, and Oxford Nanopore Technology (ONT) PromethION platform (insert size >20 kb, mean read length 22997 bp) for initial assembly, respectively.

#### 2.1.2 Genome assembly

We performed the *de novo* assembly utilizing Canu [35] and wtdbg2 (Run & Li 2020) based on ∼76 Gb high-quality ONT data. To correct the sequencing errors and improve the assembly accuracy, we performed genome sequence polishing using wtpoa-cns [69] and Pilon [86] with Illumina clean reads (∼81 Gb). The initial genome assembly was then evaluated by the Benchmarking Universal Single-Copy Orthologs (BUSCO) [76], and by the mapping rates of short reads (98.7 %) and long reads (100 %). For the chromosome-scale genome assembly, we applied the high-through chromosome conformation capture (Hi-C) technologies with library constructed from muscle tissue. Using the Illumina HiSeq platform (2 × 150 bp reads), we generated totally ∼112 Gb Hi-C clean data. After de-duplication, ∼105 million read pairs with valid interaction information were used for chromosome assembly via 3D-DNA and Juicebox [19], [20].

#### 2.1.3 Genome annotation

Based on the final assembly, the repeat elements were identified using the tandem repeats finder (TRF, [5]), LTR-FINDER [96], RepeatMasker [80], RepeatModeler [23], and *de novo* approaches. Noncoding RNAs were identified using tRNAscan-SE [44], blastn [1], and Infernal [55]. Gene prediction was conducted with incorporation of homology-based, ab initio, and RNA-Seq-assisted strategies (total RNA was extracted from five tissues muscle, skin, heart, liver, and intestine). Gene sets predicted by these three methods were then merged, and redundancy was eliminated using MAKER [12]. Final reliable gene sets were determined by integrating the outputs of MAKER and results of another commonly used annotation pipeline CEGMA [59].

#### 2.1.4 Gene functional annotation

Gene functional annotation was analyzed using the blast, KAAS [53], and InterProScan [32] searches against the public databases Nr/KOG [56], SwissProt/TrEMBL [81], KEGG [34], GO [28], Pfam [3], and AnimalTFDB [103]. Completeness of the final genome assembly and annotation was assessed by BUSCO as mentioned above.

### 2.2 Genome data of other fishes

Public genome data of other fishes were carefully selected for the subsequent comparative genomic analyses. Initially, all genomes and the corresponding annotation data available for clupeocephalan fishes (see **Introduction** for why we focused on this lineage) were downloaded from NCBI RefSeq database. Then, one fish species (genome) was selected for each order following the data-selection criteria: selecting the fish species with fundamental biological traits (e.g. metabolic rate; see below) data available first, then that with the chromosome-level genome, and finally that whose genome has highest contig N50 (usually implying best assembly). In addition, to compare with published study [39], the model species zebrafish *Danio rerio* and a closely related species of *O. bidens*, the koi *Cyprinus carpio*, were also included in the genome dataset (see **Discussion**). Finally, we obtained the genome (and the translated proteome) data for totally 12 clupeocephalan fishes, one generated in the present study, that cover ten orders (see Supplementary Table S1). Redundancy in proteomes was mitigated using CD-HIT [40]. Removal of redundancy helps to reduce the possible false-positive inference of SCO genes [78].

### 2.3 Metabolic rate data of studied fishes

We compiled the resting metabolic rate (RMR), body mass (*M*), and body temperature for each of the above 12 fish species via literature search (see Supplementary Table S1). RMR measurements of stressed fishes were not included. While raw RMR data were expressed in diverse terms such as carbon dioxide production (VCO_2_), oxygen consumption (VO_2_), and microwatts (μW), we normalized them into μW (Lighton 2008) assuming a value of 0.8 for respiratory quotient [47]. Additionally, while RMRs were measured under different body temperatures (3–26 ℃), we normalized them to 25 ℃ assuming a *Q*10 value of 1.65 [47]. Body mass was converted into unit of gram (g) of wet mass.

### 2.4 Orthology inference and gene family clustering

Orthologous gene families were clustered by OrthoVenn2 [94], which used the graph-based algorithm to identify orthologs. We performed this approach on the non-redundant proteomes (see above) with parameters *E*-value = 1e− 5 and inflation index = 2. The SCO genes (i.e. the 1:1 orthologs) presented in all 12 fishes studied in this study were extracted manually from the OthoVenn2 outputs.

### 2.5 Phylogenetic reconstruction

We employed the alignment method MUSCLE [21] to align the amino acid sequences of SCO genes. Based on the amino acid sequence alignments, we obtained the protein-coding sequence alignments via PAL2NAL [79]. Gaps and ambiguous regions were eliminated using GBlocks with default settings [13]. Species tree was then reconstructed from the concatenated protein-coding sequence alignments using maximum likelihood (ML) approach with IQ-TREE [52] and Bayesian inference (BI) with MrBayes [67]. Ultrafast bootstrap test (1000 replicates) and posterior probabilities were used to evaluate the reliability of nodes in ML and BI tree, respectively. Model selection was determined by ModelFinder [33], and the best model was GTR + F + I + G4. BI analysis was performed using two independent Markov chain Monte Carlo (MCMC) runs, each containing three hot chains and one cold chain. We discarded the first 25 % of data as burn-in to obtain the majority rule consensus BI tree. Convergence of BI analysis was assumed if the average standard deviation of split frequencies was below 0.01, and it was then further confirmed by Tracer [64]. Outgroup species was not used because preliminary results showed it didn’t affect the ingroup topology (data not shown).

### 2.6 Estimation of species divergence time of fishes

Prior to the calculation of substitution rate, a time-calibrated phylogeny was generated. We estimated the species divergence time using r8s [73] based on the species tree inferred from the concatenated protein-coding sequences of all SCO genes (see above). We ran the r8s analysis using the penalized likelihood (PL) method and TN algorithm with a smoothing parameter of one. Based on fossil data and published literature, two internal nodes of the evolutionary tree were calibrated utilizing age constraints: Ostariophysi 126.3–158.3 million years ago (Mya), Clupeocephala (i.e. the age of root node) 150.94–235 Mya [82]. Another two fossil-based data Otomorpha 150.94–228.4 Mya and Euteleostei 149–260 Mya [82] were used for results verification only.

### 2.7 Calculating the nucleotide substitution rate for each SCO gene

Rate of total nucleotide substitution was estimated using the Bayesian software package Coevol, which can account for the influence of phylogenetic non-independence [37]. The rate was reported in the absolute term (see below). While the calculation of substitution rate is generally sensitive to sequence alignments, the MUSCLE-PAL2NAL-Gblocks pipeline adopted here can facilitate creating accurate estimate [54]. With the time tree mentioned above as an input, the Coevol analysis was performed with two independent MCMC chains. Number of burn-in samples was set to default value. Chain convergence was assessed using the tracecomp program implemented within the Coevol. To guarantee good convergence, parameter difference between the two chains should be less than 0.1 and effective sample size more than 300. Absolute substitution rates were computed as Bayesian posterior instant median values, and were expressed in a unit of the expected number of substitutions over the total time from the most recent common ancestor (MRCA) down to the present [37].

### 2.8 Detection of positive selection

We utilized the branch-site model in CodeML module of PAML software [99] to identify codons putatively under positive selection in particular lineages (i.e. the foreground branches in a phylogenetic tree). The identified fast-evolving fish species was considered as foreground branch, and positive selection test was performed for each of the SCO genes separately. Briefly, genes that had undergone selection pressure were identified by the parameter omega (*ω*), which is equal to the ratio of non-synonymous (*d*_N_) to synonymous (*d*_S_) nucleotide substitution rate. An *ω* < 1 indicates purifying (or negative) selection, *ω* = 1 neutral evolution, and *ω* > 1 positive selection. We performed the likelihood-ratio test (LRT) to compare the alternative model (positive selection in specific lineages, *ω* > 1) and the null model (neutral evolution in specific lineages, *ω* = 1). The posterior Bayesian probability was calculated using the Bayes Empirical Bayes (BEB) method to assess sites under positive selection [105]. Since most of the *P*-values computed by LRT were at or very near to 1 (here about 70 % of *P*-values ≥ 0.98), the false discovery rate (FDR) correction is largely inappropriate to correct the *P*-values due to exclusion of true positives [62]. Therefore, following Potter et al. [62], significance of positive selection analyses was adopted when the unhalved LRT *P* was below 0.05 and genes should survive two additional post-hoc filtering operations. (1) Genes should have at least one BEB site with posterior probability > 0.5; (2) genes should have a median interval between BEB sites greater than 10 if the corresponding BEB site number was > 5, in order to mitigate false positives because of alignment errors [62]. Finally, we predicted and visualized the protein structure of positively-selected gene using SWISS-MODEL [88].

### 2.9 Evaluation of substitution saturation

Substitution saturation analysis was performed using the information entropy-based metric, the index of substitution saturation (*I*_ss_), implemented in the DAMBE software [92], [93]. A comparison between the *I*_ss_ value and the critical *I*_ss_ (the *I*_ss.c_) value defined a threshold for saturation in the sequences [93]. At first, we identified candidate SCO genes that might exhibit substitution saturation, which have branch length > 1 or substitutions per site > 2 [60], [74], [98]. Totally 62 such genes were determined in our data. We then ran the substitution saturation analysis for each of them. We found that for these genes all the *I*_ss_ values were smaller than the corresponding *I*_ss.c_ values (Supplementary Table S2), indicating usefulness or marginal usefulness of the sequence data. Therefore, we suggested the nucleotide substitution saturation was unlikely a problem, and all SCO genes were retained for subsequent analysis [58], [93].

### 2.10 Modeling the relationships between metabolic rate and substitution rate

When inferring the relationships between resting metabolic rate (RMR) and nucleotide substitution rate for each SCO gene, effect of body mass on RMR needs to be excluded. Generally, effects of body mass (*M*) on RMR can be described by a power-law equation [10], i.e. RMR = *a* * *M*^*b*^. We predicted the parameters *a* and *b* by taking natural logarithm of the equation, and obtained the following relationship: lnRMR = *b* * ln*M* + ln(*a*). We then calculated the adjusted RMR (equal to RMR divided by *M*^*b*^) that was considered independent of body mass. Note that the effect of body temperature on RMR had been excluded by normalization to identical temperature 25 ℃ (see Section 2.3). Parameter *b* was estimated using the phylogenetic generalized least squares (PGLS) method [27] to account for phylogenetic non-independence across species. PGLS was conducted via *nlme* package of the R environment.

For each individual SCO gene, we utilized the Coevol software to characterize the relationship between the adjusted RMR and nucleotide substitution rate with controlling for phylogenetic non-independence. Both RMR and substitution rate were analyzed in logarithmic form [36], [74]. Correlation coefficient (*r*) and posterior probability (pp) were used to describe how RMR could affect substitution rate. When pp is above 0.95, positive value of *r* indicates significantly positive relationship, and negative *r* indicates negative relationship between these two traits; otherwise (i.e. pp ≤ 0.95), there is no significant relationship, regardless of positive or negative *r* values.

### 2.11 Statistical analysis

Statistical analyses were mostly done in the R environment. Analysis of variance (ANOVA) and covariance (ANCOVA), and the Tukey’s Honest Significant Difference (HSD) test were conducted via the *stats* package.

#### 2.11.1 Identification of fast-evolving genes

This analysis was performed separately for each of the above-mentioned SCO genes presented in all twelve fish species. For a specific species (e.g. *O. bidens*), the fast-evolving SCO genes were defined as those that showed significantly higher molecular evolutionary rate in this species than in other species [24], [61]. Based on the nucleotide substitution rate estimated by Coevol with account of phylogenetic non-independence, we identified the fast-evolving genes by the non-parametric Wilcoxon signed rank test (two tailed) (following e.g. [24], [61]). Significance was adopted when the FDR-corrected Wilcoxon test *P*-value was below 0.05 (i.e. FDR < 0.05). The Wilcoxon test was also utilized to compare the fast-evolving gene numbers between different lineages, of which the correction of *P*-value (*P* < 0.05) was not necessary because it was tested just once. Wilcoxon test was conducted using the *stats* package.

#### 2.11.2 Estimates of ancestral states

For each internal node in the phylogenetic tree, ancestral state of the substitution rate was reconstructed using the Coevol software. Phylogenetic tree was rooted using the midpoint rooting method implemented in package *phytools* [65]. The midpoint rooting method produced the same tree topology with that by the outgroup rooting method (not shown).

#### 2.11.3 Phylogenetic comparative method

By treating the nucleotide substitution rate as an evolving trait itself and as a continuous variable associated with life history (see **Introduction**), we tested different evolutionary (adaptive and non-adaptive) models on substitution rate (in log10 scale) for each SCO gene using the *OUwie* package [4]. The models included Brownian motion (BM), single-regime Ornstein-Uhlenbeck (OU1), and multiple-regime OU (OUM) model. Under BM model, trait evolution follows the non-adaptive stochastic process; by contrast, under OU1 and OUM models, trait evolution follows adaptive evolutionary processes, with OU1 model fitting a single adaptive optimum whereas OUM model fitting multiple optima. Model performance was compared by the LRT *P*-value corrected by FDR (FDR < 0.05).

#### 2.11.4 Gene set enrichment analysis

Gene ontology (GO) and functional enrichment analyses for SCO gene subsets (e.g. fast-evolving genes) were performed using the Database for Annotation, Visualization and Integrated Discovery (DAVID) [31]. We used all the SCO genes as background. Significant GO terms were determined according to *P*-value with correction by FDR (FDR < 0.05).

## 3 Results

### 3.1 Genome of the Chinese hook snout carp *O. bidens*

**Fig. 1:**
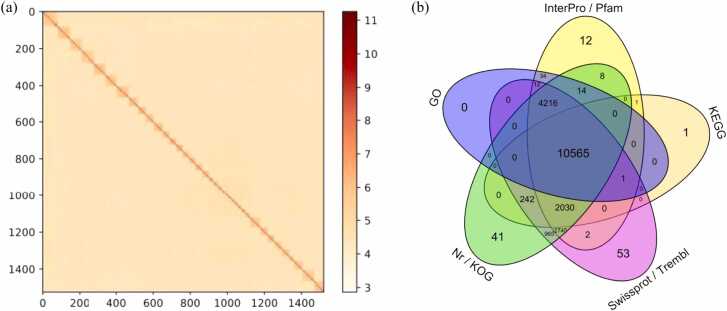
Characterization of the *Opsariichthys bidens* genome reported in this study. (a) Hi-C heatmap of chromosomal contacts with 500k resolution. Blocks represent the pseudochromosomes. Color indicates the contact density, with lighter color for lower values and darker color for higher values. The coordinates indicate the number (N, in Mb) on the genome multiplied by bin size (i.e. N * bin). (b) Function annotations of the protein-coding genes by different databases (see Methods).

Chromosome-level genome was determined for the Chinese hook snout carp *O. bidens*, using the short-read Illumina HiSeq, long-read Oxford Nanopore PromethION, and Hi-C technologies (Fig. 1). The ultimate genome assembly comprised 787.13 Mb (96.6 % of which were anchored into 38 pseudochromosomes), and has a contig N50 of 6.50 Mb and scaffold N50 of 22.82 Mb (Supplementary Table S3). In total, 21375 protein-coding genes were predicted in the *O. bidens* genome, with 98.0 % of these genes hitting the record in at least one of the databases such as InterPro, GO, KEGG, Nr, Swissprot (Supplementary Table S3, Fig. 1b). BUSCO analysis showed that 91.9 % of the single-copy core genes across ray-finned fishes (superclass Actinopterygii) were completely present in the *O.bidens* genome, indicating a relatively complete annotation set. Overall, *O. bidens* had an average gene size of 19.75 kb, protein-coding sequence length of 1.71 kb, and intron length of 1.73 kb (Supplementary Table S3). There were about 10.66 exons (mean length 280.7 bp) per gene. It’s estimated that about 53 % (418.13 Mb) of the genome were the repeat elements, while 0.08 % (0.66 Mb) were non-coding RNAs (miRNA, tRNA, rRNA, and snRNA) (Supplementary Table S3).

### 3.2 Variation in substitution rate for genes at the genome-wide level

**Fig. 2:**
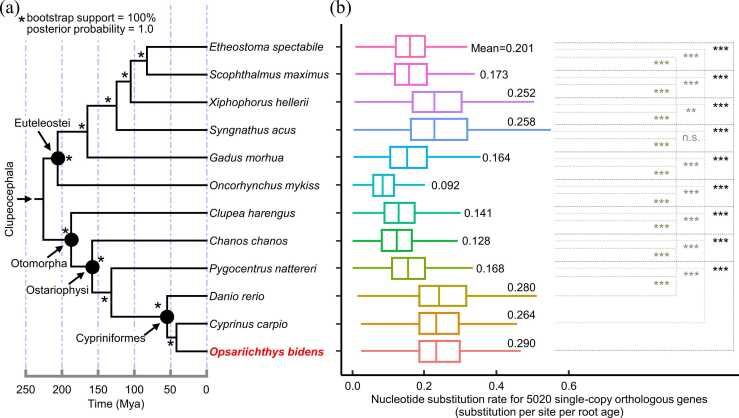
The inferred nucleotide substitution rate for genes at the genome-wide level across twelve clupeocephalan fishes. (a) Time tree of the studied fishes with the one sequenced in this study being marked in red (the Chinese hook snout carp *Opsariichthys bidens*). Mya: million years ago. Bootstrap support and posterior probability values are obtained using the maximum likelihood and Bayesian approach, respectively (see Methods). (b) Boxplot of nucleotide substitution rate of 5020 single-copy orthologous genes (with outliers not shown). The substitution rate is expressed in substitutions per site per root age, with the root age equal to 225.84 million years. Substitution rate is estimated by Coevol that has accounted for phylogenetic non-independence. Also shown are the results of multiple pairwise comparisons between one cypriniform fish and one non-cypriniform fish (**, adjusted *P* < 0.01; ***, adjusted *P* < 0.001; n.s., not significant). Numbers at the right side of the box indicate the mean substitution rate values of each species.

We identified totally 5020 SCO genes (an extra SCO gene was removed due to lack of consensus alignment) presented in all studied fishes. Phylogeny based on the concatenated protein-coding sequences of these genes supported a split between cohorts Euteleostei and Otomorpha, and recovered the monophyly of subcohort Ostariophysi (carps, milkfish, piranha, etc.) (Fig. 2a). Regarding the molecular evolution rate for SCO genes, we observed substantial variations in total nucleotide substitution rate (estimated by Coevol) across both species and genes (Fig. 2b). The chain convergence of Coevol analysis was good and displayed in Supplementary Fig. S1. For a given SCO gene, the cross-species substitution rate showed on average 10.88-fold difference between the lowest and the highest values. For a given species, the cross-gene substitution rate showed on average 641.66-fold difference between the lowest and the highest values. Because phylogenetic non-independence had been accounted for by Coevol, we utilized ANOVA analysis to do group comparison on the nucleotide substitution rate. As a result, we detected significant variations in substitution rate among species groups (ANOVA *P* < 2.0e−16). Subsequent multiple pairwise comparisons indicated that the SCO genes from three species (*O. bidens* sequenced in the present study, zebrafish *Danio rerio*, and koi *Cyprinus carpio*) evolved relatively faster than those from the remaining fishes (mean 0.264–0.290 versus 0.092–0.258 substitutions / site / root age) (Supplementary Table S4). Namely, there were 26 out of 27 pairwise comparisons (one of the above three fishes versus one of the remaining fishes) supporting significantly higher substitution rate in the three fish species (Tukey’s HSD test, adjusted *P* < 0.01) (Fig. 2b, Supplementary Table S4). All these three fast-evolving species belong to the order Cypriniformes (Fig. 2). Within this order, *O. bidens* evolved significantly faster than *C. carpio* (adjusted *P* < 0.001) and marginally significantly faster than *D. rerio* (adjusted *P* = 0.06).

### 3.3 Fast-evolving genes in *O. bidens*

**Fig. 3:**
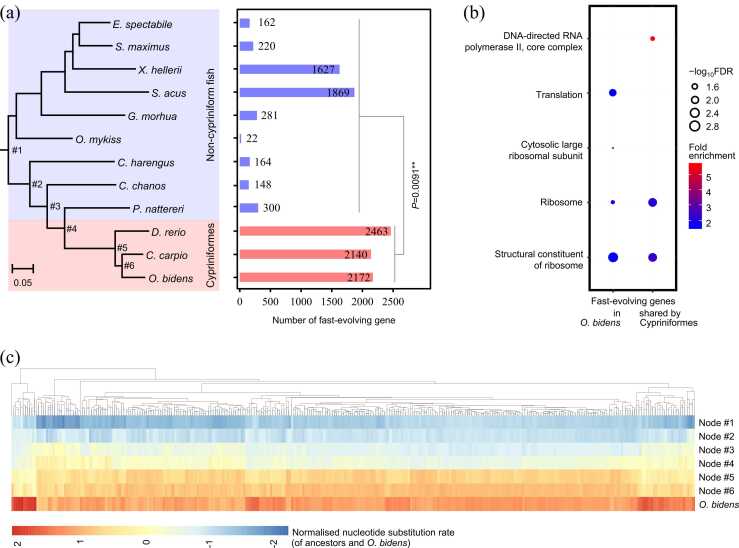
Fast-evolving genes in the Chinese hook snout carp *O. bidens*. (a) *O. bidens* and two related cypriniform fishes have significantly more fast-evolving genes than the remaining fishes. * * indicates *P* < 0.01. The evolutionary tree is reconstructed by a maximum likelihood method based on concatenated matrix of single-copy orthologous genes. All nodes are supported with 100 % bootstrap values. Label at a node in the tree represents the node IDs. Scale bar indicates the nucleotide substitutions per site. (b) GO enrichment of all the 2172 fast-evolving genes identified in *O. bidens* and the 741 fast-evolving genes shared by three cypriniform fishes (not found in any of the rest fishes, see the main text). Shown are the statistics of significantly enriched (FDR < 0.05) GO terms. (c) Heatmap showing the 581 genes exhibiting a constant trend towards increased substitution rate in the *O. bidens* compared with the root. More than 94 % (547/581) of these genes are identified as fast-evolving genes in *O. bidens*. Rows of the heatmap represent the internal nodes or tip (*O. bidens*) in the fish tree, and the internal node IDs are the same as those shown in (a). Genes (i.e. columns of the heatmap) are grouped using an unsupervised hierarchical clustering algorithm. Substitution rates are centered and scaled in the column direction, with red color indicating large value and blue color small value.

More fast-evolving SCO genes were identified in the above-mentioned three species *O. bidens*, *D. rerio*, and *C. carpio* than the remaining fish species: 2140–2463 versus 22–1869 or in percentage 42.6–49.1 % versus 0.4–37.2 % (mean 2258 versus 533, Wilcoxon test *P* = 0.009) (Fig. 3a). GO enrichment analysis (FDR < 0.05) showed that these fast-evolving genes were mainly enriched in ribosome and translation process (with *O. bidens* as an example, Fig. 3b). Note that nearly half of all SCO genes were identified as fast-evolving genes for *O. bidens* (Fig. 3a). Totally 741 fast-evolving genes were shared by cypriniform fishes *O. bidens*, *D. rerio*, and *C. carpio*, but not by any of the remaining fishes. These (741) genes were significantly (FDR < 0.05) enriched in several GO terms related to ribosome and DNA-directed RNA polymerase II core complex (Fig. 3b). Another 180 fast-evolving genes were found to be exclusive to *O*. *bidens*. No significant GO terms were found (FDR > 0.05) for these (180) genes exclusive to *O. bidens*, suggesting their involvement in a range of biological functions.

### 3.4 Ancestral states of fast-evolving genes in *O. bidens*

To compare the molecular evolution rate between extant fishes with the ancestors, we reconstructed the ancestral states of the nucleotide substitution rate for the eleven internal nodes on the fish tree. In particular, we focused on genes that displayed constant trends of substitution rate alteration from the most recent common ancestor (MRCA) down to the present. As a result, we found that among all the 5020 SCO genes there were 578–596 (mean 585) genes exhibiting constant trend towards increased substitution rate for the above-mentioned three species *O. bidens*, *D. rerio*, and *C. carpio*. By contrast, the corresponding gene number was significantly smaller, 22–678 (mean 256) for the remaining fishes (ANCOVA *P* = 0.0035 with the number of internal nodes as covariate). The 581 such genes in *O. bidens* showed a mean 2.22-fold increase relative to the MRCA (Fig. 3c). We noticed that most (547/581, 94.1 %) of these genes can be also identified as fast-evolving genes in *O. bidens*, which constituted a proportion of 24.9 % (541/2172) of all the fast-evolving genes identified in *O. bidens*. This indicated an ancestral contribution to the increase in nucleotide substitution rate of the Chinese hook snout carp (Fig. 3c).

### 3.5 Positive selection of the fast-evolving genes in *O. bidens*

**Fig. 4:**
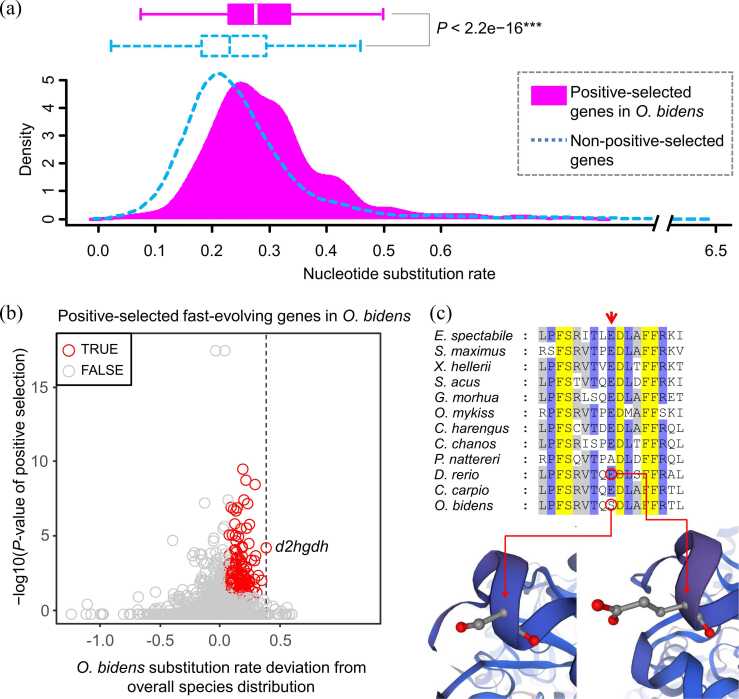
Positive selection analysis of the fast-evolving genes identified in the Chinese hook snout carp *O. bidens*. (a) Boxplot and density distribution curve showing the difference in nucleotide substitution rate between the positive-selected genes and non-positive-selected genes. The substitution rate is expressed in nucleotide substitutions per site per root age, with the root age equal to 225.84 million years. Also shown is the *P*-value of Wilcoxon test (*** indicating *P* < 0.001). (b) Fast-evolving genes have experienced positive selection in *O. bidens*. The substitution rate deviation from overall species distribution is defined as the substitution rate of *O. bidens* minus the upper value of 95 % confidence interval (CI) of all studied species. TRUE: the fast-evolving genes that have undergone positive selection (i.e. the positive-selected fast-evolving genes, see the main text) in *O. bidens*; FALSE: other genes. Possibly important candidate gene *d2hgdh* (see the main text) is labeled with text. Dashed line highlights the position of *d2hgdh* gene on the x axis. (**c**) Partial sequence alignment and 3D protein structure mapping show the positively selected site 76 in *d2hgdh* gene of *O. bidens*. Red arrow above the sequence indicates the location of site 76. At this site, while glutamic acid (one-letter abbreviation E) was present in most of other species, serine (S) was present in species *O. bidens*. This E to S substitution (i.e. E76S) was a reflection of *O. bidens* undergoing positive selection (see the main text). The residue 76E and 76S was also marked on the D2HGDH protein structure of *O. bidens* and zebrafish *D. rerio* (a representative of other species), respectively.

With the 0.05 threshold of LRT *P*-values and additional post-hoc filtering (see **Methods**), we detected 316 SCO genes that had experienced selection pressure in *O. bidens*. Compared with the non-positive-selected genes in *O. bidens*, the positively selected ones showed significantly faster rate of nucleotide substitution (Wilcoxon test *P* < 2.2e−16) (Fig. 4a), indicating a connection between positive selection and the accelerated molecular evolution of this fish species.

By comparing the positive-selected genes identified among the three cypriniform species, we found that 258 positive-selected genes (81.6 % of the 316 genes) were exclusive to *O*. *bidens* and not shared by either *C*. *carpio* or *D*. *rerio*. More importantly, 166 positive-selected genes (52.5 % of 316 genes) were concurrently identified as fast-evolving genes in *O. bidens* (Fig. 4b). We termed these 166 genes the positive-selected fast-evolving genes. They included the RNA helicase genes *ddx46*, *ddx52*, *dhx30*, *dhx38*, *dhx57*, and the gene *ercc6*. By integrating the results of positive selection and the above identification of fast-evolving genes exclusive to *O. bidens*, we were able to detect the positive-selected fast-evolving genes that are specific to this fish species. We noticed that one such gene (*O. bidens* positive selection *P* = 3.4e−5, fast evolution FDR = 0.0066), the D-2-hydroxyglutarate dehydrogenase gene *d2hgdh* of *O. bidens*, deviated most from the distribution of nucleotide substitution rate of overall species, when compared with other positive-selected fast-evolving genes (Fig. 4b). In Fig. 4c, we showed the positively selected site in *d2hgdh* gene (site 76) with the greatest value of BEB posterior probability (0.998). At this site, natural selection was responsible for the substitution of glutamic acid (negatively charged amino acid) to serine (uncharged amino acid) in species *O. bidens* (E76S substitution) (Fig. 4c). This site was located in the α-helix close to the N-terminal of D2HGDH protein (Fig. 4c).

### 3.6 Evolutionary modes of the fast-evolving genes in *O. bidens*

As mentioned above, there were 741 and 180 fast-evolving SCO genes that were shared by three cypriniform fishes (*O. bidens*, *D. rerio*, and *C. carpio*) and specific to *O. bidens*, respectively. These genes were not rapidly evolving in any of the non-cypriniform fishes. To determine the evolutionary mechanisms associated with the divergence in substitution rate among fishes, we compared and evaluated three evolutionary models for each of these genes: the Brownian motion (BM), single-regime Ornstein-Uhlenbeck (OU) (OU1), and multiple-regime OU (OUM) model. While BM model incorporates a non-adaptive random evolutionary process, both OU1 and OUM models depict an adaptive evolutionary process: OU1 model assuming trait changes towards a single optimum, OUM model allowing optima to vary across lineages in the phylogeny.

**Fig. 5:**
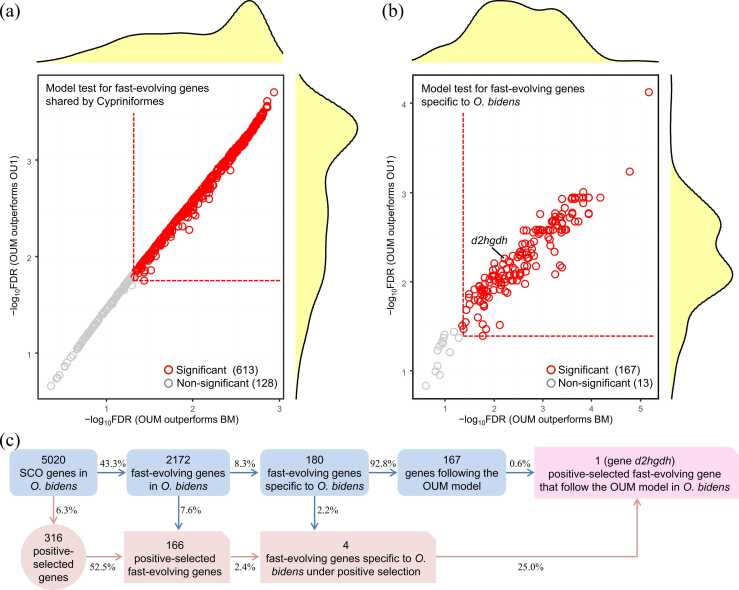
Evolutionary model fitting on the nucleotide substitution rate of the fast-evolving genes. (a) Model fitting for fast-evolving genes shared by three cypriniform fishes (these genes are not identified as fast-evolving genes in any of the remaining nine fishes). The curves show the density distribution of log-transformed FDR values (i.e. the −log_10_FDR values). BM: Brownian motion model; OU1: single-regime Ornstein-Uhlenbeck (OU) model; OUM: multiple-regime OU model. Each data point represents a gene. Significance is determined when the OUM model outperforms both BM and OU1 models (FDR < 0.05). (b) Model fitting for fast-evolving genes specific to *O. bidens* (these genes are not identified as fast-evolving genes in any of the remaining 11 fishes). Possibly important candidate gene *d2hgdh* (see Fig. 4b and the main text) is labeled with text. (**c**) Summary of adaptive analyses on carp *O. bidens* showing gene *d2hgdh* is the only positive-selected fast-evolving gene specific to *O. bidens* and concurrently following the OUM evolutionary model.

We found that OUM model fitted best for each of these genes, and significance was reached for 82.7 % (613/741) and 92.8 % (167/180) of the fast-evolving genes shared by three cypriniform fishes (1.8e−4 < FDR < 0.05; Fig. 5a) and specific to *O. bidens* (7.3e−6 < FDR < 0.05; Fig. 5b), respectively. By integrating the results of OUM model fitting and positive selection analyses, we found the evolution of the candidate gene *d2hgdh*, which was the positive-selected fast-evolving gene specific to *O. bidens* (Fig. 4b), followed the OUM model: FDR = 0.0066 for OUM outperforming BM model and FDR = 0.0052 for OUM outperforming OU1 model (Fig. 5b). Indeed, among the 180 fast-evolving genes specific to *O. bidens*, the *d2hgdh* gene was the only one that had evolved under the OUM model and concurrently experienced positive selection (Fig. 5c). Together, these results verified the nucleotide substitution rate variation from an evolutionary perspective, i.e. the variation was a reflection of different lineages being attracted to different adaptive optima, suggesting adaptive evolution rather than random drift contributed to the fast molecular evolution of these genes in *O. bidens*.

### 3.7 Gene-specific effects of resting metabolic rate (RMR) on molecular evolution rate

**Fig. 6:**
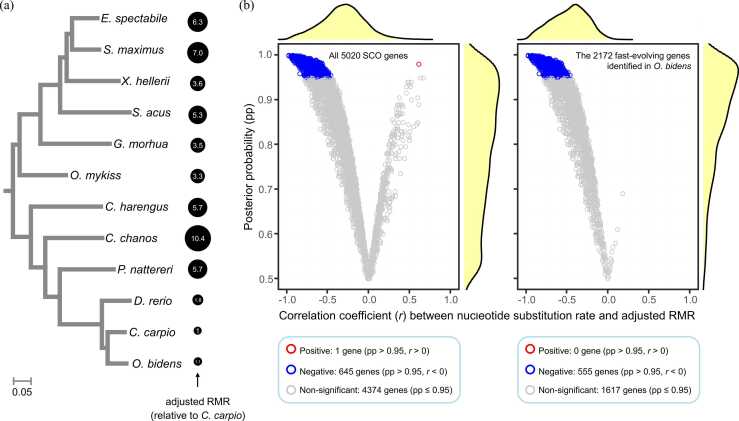
Effects of resting metabolic rate (RMR) on nucleotide substitution rates of 5020 single-copy orthologous genes. (a) The Chinese hook snout carp *O. bidens* and two related cypriniform fishes have relatively lower RMR than the remaining fishes (the smaller the circle the lower the RMR). The adjusted RMR represents RMR with correction for influence of body mass, body temperature, and phylogenetic non-independence (see Methods). Numbers in circles indicate the adjusted RMR of a species divided by that of *C. carpio* (the lowest-RMR species). The evolutionary tree is a maximum-likelihood tree same as that in Fig. 3a. All nodes are supported with 100 % bootstrap values. Scale bar indicates the nucleotide substitutions per site. (b) Estimates of relationship between the nucleotide substitution rate and adjusted RMR for each of all 5020 single-copy orthologous (SCO) genes (left panel) and 2172 fast-evolving SCO genes identified in *O. bidens* (right panel). Each data point represents a gene. Values of posterior probability (pp) and correlation coefficient (*r*) are estimated within the Bayesian framework via Coevol with accounting for phylogenetic non-independence. Red, blue, and gray color indicates significantly positive (pp > 0.95, *r* > 0), significantly negative (pp > 0.95, *r* < 0), and non-significant relationship (pp ≤ 0.95), respectively. The curves show the density distribution of pp (vertical plot) and *r* (horizontal plot) values.

Fishes studied here covered a body mass range of 0.481–6650 g, body temperature of 3–28 ℃, and RMR of 266.71–724185 µW (Supplementary Table S1). With accounting for effects of body mass, body temperature, and phylogenetic non-independence on RMR (see **Methods**), we obtained the adjusted RMR, which showed a 10.4-fold difference between the lowest-RMR species (koi *C. carpio*) and the highest-RMR species (the milkfish *Chanos chanos*) (Fig. 6a).

In line with expectation, the relationship between nucleotide substitution rate and the adjusted RMR varied greatly across genes, according to Coevol analysis. At the 0.95 threshold of Coevol posterior probability (pp), totally 4374 (87.1 % of all 5020) SCO genes, including 1617 (74.5 % of 2172) fast-evolving genes identified in *O. bidens*, showed no significant relationships (pp ≤ 0.95, see **Methods**) between nucleotide substitution rate and adjusted RMR (Fig. 6b). Only one SCO gene (basic helix-loop-helix family member e23 *bhlhe23*) showed significantly positive relationship (pp = 0.98, correlation coefficient *r* = 0.61) (Fig. 6b), and this gene was not a member of fast-evolving genes identified in *O. bidens*. By contrast, 645 (12.9 %) of all 5020 SCO genes exhibited significantly (pp > 0.95) negative relationship between nucleotide substitution rate and adjusted RMR (mean pp = 0.98, mean *r* = −0.72) (Fig. 6b). Importantly, 555 of these significantly negative-correlated genes can be concurrently identified as fast-evolving genes, constituting a proportion of 25.6 % (555/2172) of all the fast-evolving genes identified in *O. bidens*. We found that such 555 genes were significantly (FDR < 0.05) enriched in GO terms related to ribosome and translation, the same as those displayed in Fig. 3b. We also noticed that 46 of the negative-correlated genes had experienced position selection in *O. bidens*; however, no significant GO terms were detected (FDR > 0.05) for these genes.

## 4 Discussion

### 4.1 Rapid evolution manifests in SCO genes at the genome-wide level in *O. bidens*

Fast substitution rate has been widely reported among not only lineages but also genes (e.g. [60], [68], [42], [91]). However, this question has been less investigated for genes at the genome-wide level across lineages. In the present work, using comparative genomic analysis on twelve clupeocephalan fishes including the newly-sequenced fish *O. bidens*, we uncovered the mode of variation in nucleotide substitution rate for 5020 single-copy orthologous (SCO) genes. Our results revealed great variations between both species and genes, suggesting *O. bidens* and other clupeocephalan fishes can serve as good systems to explore fast molecular evolution.

We found that *O. bidens* (as well as *C. carpio* and *D. rerio*) displayed significantly higher substitution rate averaged from SCO genes than almost all the remaining fishes studied here (Fig. 2). This is in agreement with the previous observation that zebrafish *D. rerio* exhibited fast molecular evolution according to analysis on the concatenated matrix of orthologous non-coding sequences from four fishes (Lee et al., 2010). These three fast-evolving species (from three families) all belong to order Cypriniformes, implying that fast molecular evolution might be an order-level characteristic. However, since only 12 representative species were compared in the present study (to satisfy the data-selection criteria, see **Methods**), this supposition requires further investigation in future work using more species and genome data. If that’s really the case, the high substitution rate of this order may be associated with its high species richness [102]. More importantly, we presented a previously unrecognized manifestation of rapid evolution at the molecular level. That is, we found a large proportion (42.6–49.1 %) of SCO genes were identified as fast-evolving genes in *O. bidens*, koi *C. carpio* and zebrafish *D. rerio*; by contrast, the proportion (0.4–37.2 %) was significantly smaller in the remaining fishes (Fig. 3).

We showed that within the three cypriniform species, *O. bidens* evolved significantly or marginally significantly faster than *C. carpio* and *D. rerio*. It may be expected that fast evolution is reflected in specific genes associated with specific functions such as environmental response or reproduction [51], [83], [91], [98]. In contrast to this hypothesis, our results showed the rapidly evolving genes (2172 genes in *O. bidens*) were not necessary to be limited to particular gene groups, and here they were involved in a variety of functions and even several very basic biological processes such as translation and ribosome that are critical for biological fitness. This is in line with previous work that highly critical and conserved genes can also evolve faster, e.g. the 18 S ribosomal gene [24] and centromeric proteins [68]. Taken together, our observations suggest the rapid evolution is indicative of wide genetic effect in the genome of *O. bidens*.

### 4.2 Evolutionary and ecological factors contributing to the fast substitution rate of *O. bidens*

It is challenging to address why the accelerated substitution rate would happen, which has been argued a lot during the past decades in evolutionary biology (see **Introduction**). Our results provide several explanations for the fast rate of nucleotide substitution observed in *O. bidens*.

First, previously understudied contributions from the ancestors to the elevated substitution rate. We found that about a quarter (25 %) of the fast-evolving SCO genes identified in *O. bidens* exhibited a constant increase of substitution rate from the MRCA down to the present (Fig. 3c), suggesting the non-negligible contribution from the ancestral states. This possibly implies the existence of some historical events and/or persistent mechanisms in affecting the nucleotide substitution (through mediating the DNA replication, mutation, and repair) of these genes. For instance, one such mechanism might be that the DNA repair machinery was not working properly in the very early clupeocephalan fishes, which resulted in a burst of increased substitution rate of many genes [90]. During the subsequent species diversification, the DNA repair efficiency may also diverge and vary across different lineages [36]. Consequently, the substitution of these genes in *O. bidens* ancestors was further accumulated, possibly due to a constantly reduced efficiency of DNA repair. To the best of our knowledge, although many factors have been proposed to explain the substitution rate variations [42], [50], [71], [74], whether and how such variations are relevant to ancestral states has so far received limited attention for individual genes from a genome-wide perspective. The present study may thus be a pioneer attempt for future studies in this regard. We also noticed that different genes can have distinct magnitudes of increase in substitution rate compared with the MRCA, which appears to be a reflection of different evolutionary histories due to selection pressure, horizontal gene transfer, etc. These genes may provide a resource for exploring the heterogeneous and gene-specific effects from the ancestors.

Second, adaptive selection contributes to the increased substitution rate. Although it is not an unusual view that high rate of molecular evolution may reflect adaptive selection (e.g. [26], [87], [30], [45], [91]), non-adaptive neutral drift as an alternative explanation has also been proposed since long ago (e.g. [104], [2], [17]). Given that molecular evolution rate is an evolving trait itself linked with life history (see **Introduction**), we compared these two evolutionary mechanisms (adaptation and non-adaptive drift) through phylogenetic comparative analysis that is widely used on life-history traits to evaluate their contributions to fast molecular evolution. Our results provide strong support for the adaptation assumption. We showed that for most (> 80 %) of the fast-evolving genes that were shared by the three cypriniform fishes (*O. bidens*, *C. carpio* and *D. rerio*) and specific to *O. bidens*, the substitution rate followed the adaptive model OUM rather than the OU1 or random-drift BM models (FDR < 0.05) (Fig. 5). In other words, the increased substitution rate of these genes is a reflection of evolution towards an elevated adaptive optimum [4]. Additionally, we found that the positive-selected genes showed significantly higher substitution rate than non-positive-selected genes in *O. bidens*, and 166 fast-evolving genes identified in this species had concurrently experienced positive selection (Fig. 4). Our results indicate that the fast-evolving genes can be under selective pressures, and adaptive selection did influence the accelerated evolution [98]. We noticed that among the 180 fast-evolving genes specific to *O. bidens*, only one gene (*d2hgdh*) under positive selection followed the OUM model (Fig. 5c), which indicates a relatively low overlap between these two adaptive analyses. This is due to that the OUM model fitting analysis is based on substitution rate itself (i.e. all substitutions from all sites regardless of non-synonymous or synonymous substitutions), whereas positive selection analysis focuses on just a single or a small subset of sites with more non-synonymous than synonymous substitutions. Nevertheless, since positive selection is often linked with structural and thus functional alternation of proteins, these positively-selected genes may be functionally divergent in the studied fishes [100], [26].

Finally, the resting metabolic rate (RMR) can affect the nucleotide substitution rate in a gene-specific manner. Overall, our results reveal low metabolic rate in *O. bidens* and its closely related species (*C. carpio* and *D. rerio*) in comparison to other clupeocephalan fishes studied here (Fig. 6a), thus confirming previous observation [16]. In contrast to the original statement of ‘metabolic rate hypothesis’ (e.g. [49], [25]), we found RMR exerted a gene-specific rather than a universal positive effect on substitution rate (although this may need to be verified by more species and rigorous experimental studies). Indeed, with controlling for the influence of body mass, body temperature, and phylogenetic non-independence, we showed that the relationship between the adjusted RMR and nucleotide substitution rate could be either positive, absent, or even negative in different genes (Fig. 6). In particular, we found that 25.55 % (555/2172) of fast-evolving genes identified in *O. bidens* exhibited significantly negative correlations between substitution rate and adjusted RMR. This observation echoes a previous study on a lineage within Clupeocephala (scombroids) which revealed that scombroids with low metabolic rate showed a high substitution rate [63].

Three possible mechanisms are proposed to address the gene-specific relationship observed here between metabolic rate and molecular evolution rate. (i) RMR is generally supposed to be linked to substitution rate via influencing the DNA damage [49]; however, DNA repair efficiency in response to DNA damage varies across genomic regions and genes owing to differences in chromatin structure, DNA modification, nucleotide composition, etc. [2]. It is thus unlikely that an increase in rate of DNA damage due to high RMR will result in a proportionate increase in substitution rate [36]. (ii) Different genes can have distinct evolutionary histories that differ from one another because of selection pressure, horizontal gene transfer, etc. Such differences could reflect as variations in substitution rate and therefore eliminate the universal relationship. (iii) Besides metabolic rate, other factors such as many life-history traits and environmental variables could also impact the substitution rate [2], [42], [50], [70], [71], [74], [89], [9]. This may confound any single relationship between RMR and substitution rate, especially for those genes involved in these factors. Notably, many of these factors, e.g. body mass, generation time, longevity, and latitude, are known to exert negative influence on substitution rate [50], [63], [71], [74], [9] and concurrently to covary with metabolic rate [10]. This probably provides reasonable explanations why many fast-evolving genes identified here exhibit significantly negative (rather than positive) correlation (Fig. 6): it might be a consequence of both the adjusted RMR and substitution rate being affected by other untested variables. This could deserve further considerations.

### 4.3 Molecular insights into the adaptation and evolution of *O. bidens*

As one of the most widely distributed cyprinids in eastern Asia, the Chinese hook snout carp *O. bidens* shows adaptation capacity to both the field and industrial farming environments, which is possibly due to its high hypoxia tolerance [101], [14]. If this has genetic underpinnings, adaptation and/or positive selection should be detected at molecular level. In line with this supposition, we find many genes that have experienced positive selection and fast evolution in *O. bidens*. These include genes (e.g. RNA helicase genes) that involve in response to environmental stress [11], [84], [85]. In addition, the positive-selected fast-evolving gene *d2hgdh* (Fig. 4, Fig. 5) specific to *O. bidens* encodes the enzyme D2HGDH, which is associated with glycolytic capacity that is critical for metabolic adaptation to hypoxia [57]. We showed that the E76S substitution detected in *O. bidens d2hgdh* gene was driven by positive selection (Fig. 4c). Importantly, this positively selected site was located in the first α-helix of FAD-binding domain of D2HGDH [97], thus implying the E76S substitution may involve in functional alteration associated with adaptation of *O. bidens*.

Besides these individual genes, several genome-level insights can be gained to explore the molecular evolution of *O. bidens* and clupeocephalan fishes. For instance, genome GC content of *O. bidens* was one of the lowest values among the fish species studied here (Supplementary Table S1), and this low GC content may be linked to the organism-level low RMR, given the significant positive correlation between these two traits (*r* = 0.66, *P* = 0.02). By contrast, negative (and non-significant) correlation exists between RMR and genome size (*r* = −0.43, *P* = 0.16), which appears to support the hypothesis that high metabolic rate is associated with reduced genome size [29]. In the genomic era, genome-scale data is widely used to address ambiguous relationships; however, it can also cause contradictions [72], [75], [8]. The Clupeocephala tree obtained in our study (Fig. 2a) is consistent with previous observations, such as the split between cohorts Otomorpha and Euteleosteiis, and the monophyly of Ostariophysi [7], [18]. Likewise, the estimated species divergence dates are compatible with two independent fossil-based data (Otomorpha 150.94–228.4 and Euteleostei 149–260 Mya) (Fig. 2a). On this account, our results support that phylogenomics is a good choice for the inference of tree of clupeocephalan fishes.

Our study also offers insights into the chromosome evolution of *O. bidens*. Previous genomic studies showed that the haploid chromosome number of this species varied between sexes, e.g. females had 39 chromosomes while males 38 chromosomes [43], [95]. However, in our study, the female *O. bidens* also carried 38 chromosomes, suggesting sex may be not the major determinant of chromosome number variation. The evolution of *O. bidens* chromosome is complex, in which some factors like the geographic location may also play important roles [41]. Therefore, our study provides a resource for future investigations to further explore this question, and contribute to understanding the fast evolution and adaptation of *O. bidens* from a molecular perspective.

## 5 Conclusions

Our results indicate that compared with most of the fishes studied here, *O. bidens* displays a fast rate of molecular evolution at the genome-wide level. This is reflected as not only the significantly higher mean value of substitution rate of 5020 SCO genes (0.29 substitutions per site per root age) but also the greater number of fast-evolving genes (2172 genes). We conjecture that the fast molecular evolution is likely a common characteristic of the three cypriniform fishes *O. bidens*, koi *C. carpio*, and zebrafish *D. rerio*. We provide evidence for the view that rapid molecular evolution may occur in highly conserved DNA, because the fast-evolving genes identified in *O. bidens* are associated with very basic biological processes and enriched in functionally significant genes involving translation and ribosome that are critical for biological fitness. These observations indicate a wide genetic effect of rapid evolution in the *O. bidens* genome.

With regards to the evolutionary and ecological factors relevant to the rapid substitution rate for genes, we quantitatively explored an important but previously underemphasized contribution from ancestral states. We show that the adaptive evolutionary model rather than random-drift model fits the substitution rate best for ∼85 % (780/921) of the fast-evolving genes specific to *O. bidens* and related species, and 7.6 % (166/2172) of fast-evolving genes have experienced positive selection in *O. bidens*. We present a gene-specific effect of resting metabolic rate (RMR) on substitution rate. Such a heterogeneous effect of RMR may be attributed to both the biological/evolutionary differences among genes and the confounding influence of potentially covarying variables. We provide suggestions for future studies on the relationships between other untested life-history traits and fast molecular evolution, and on the role of candidate genes (e.g. *d2hgdh*) in adaptive evolution of *O. bidens* at the molecular level.

## CRediT authorship contribution statement

**Wei Wang:** Conceptualization, Data curation, Formal analysis, Investigation, Methodology, Validation, Visualization, Writing – original draft, Writing – review & editing. **Fengbo Li:** Data curation, Investigation, Methodology, Resources, Supervision, Writing – review & editing. **Ming Li:** Investigation, Resources. **Haihua Cheng:** Investigation.

## Declaration of Competing Interest

The authors declare that they have no competing interests.

## Data Availability

Final genome assembly of the Chinese hook snout carp has been submitted to NCBI GenBank database under the BioProject number PRJNA792918. Data of 5020 single-copy orthologous gene sequence alignments as well as GO terms, and fast-evolving gene lists, etc. are deposited at FigShare (10.6084/m9.figshare.25854061).
