# Supplementary material for: Genome-wide analysis reveals the contributors to fast molecular evolution of the Chinese hook snout carp (Opsariichthys bidens)
Source: Comput Struct Biotechnol J. 2024 May 31;23:2465–77. doi: 10.1016/j.csbj.2024.05.048 (PMC11179538; doi:10.1016/j.csbj.2024.05.048)
Supplement: Supplementary file 1 — Supplementary material [file mmc1.pdf]

Supplementary Figure S1. Convergence assessment of the MCMC chains in Coevol analysis. For each Coevol parameter (e.g. lnL), effective sample size greater than 300 and difference between chains lower than 0.1 indicate good convergence. All parameters fulfilled the criteria. Each data point represents a gene.

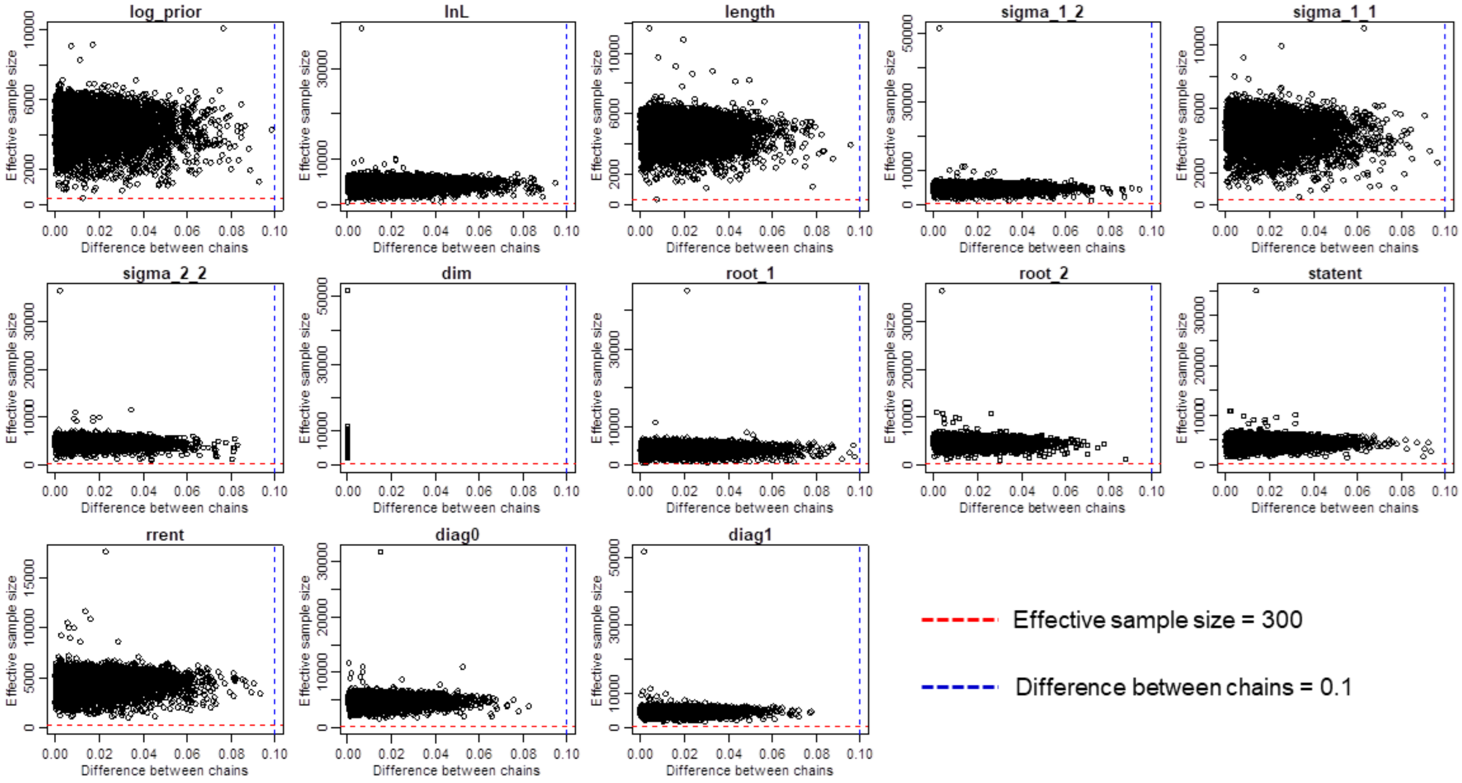

Supplementary Table S1. Fish genome and resting metabolic rate (RMR) data used in the present study. Species are sorted and displayed according to their ordinal names.

Species	Common name	Order	Family	NCBI genome accession	Genome size (Mb)	GC content (%)	Body mass (g)	Body temperature (℃)	RMR (μW)	References
Pygocentrus nattereri	red-bellied piranha	Characiformes	Serrasalmidae	GCF_015220715.1	1222	40.7	167.5	27	148844.9	Duncan 2020
Clupea harengus	Atlantic herring	Clupeiformes	Clupeidae	GCF_900700415.2	786	44.2	128	9.3	48366.02	Johnstone et al. 1993
Cyprinus carpio	koi	Cypriniformes	Cyprinidae	GCF_018340385.1	1680	37.2	174	10	11501.4	Makarieva et al. 2008
Danio rerio	zebrafish	Cypriniformes	Danionidae	GCF_000002035.6	1373	36.7	0.481	28	266.7127	Barrionuevo & Burggren 1999
Opsariichthys bidens	Chinese hook snout carp	Cypriniformes	Xenocyprididae	This study	787	38.1	4.6145	26	1085.032	Fu et al. 2012
Xiphophorus hellerii	green swordtail	Cyprinodontiformes	Poeciliidae	GCF_003331165.1	733	41.2	2	25	1665	Makarieva et al. 2008
Gadus morhua	Atlantic cod	Gadiformes	Gadidae	GCF_902167405.1	670	45.8	6650	3	724185	Makarieva et al. 2008
Chanos chanos	milkfish	Gonorynchiformes	Chanidae	GCF_902362185.1	657	41.6	0.7	25	1884.33	Makarieva et al. 2008
Etheostoma spectabile	orangethroat darter	Perciformes	Percidae	GCF_008692095.1	855	41.0	12.35	17.5	10088.72	Makarieva et al. 2008
Scophthalmus maximus	turbot	Pleuronectiformes	Scophthalmidae	GCF_013347765.1	557	43.4	142	12.5	76027.88	Brown et al. 1984
Oncorhynchus mykiss	rainbow trout	Salmoniformes	Salmonidae	GCF_013265735.1	2342	43.5	450	5	68265	Makarieva et al. 2008
Syngnathus acus	greater pipefish	Syngnathiformes	Syngnathidae	GCF_901709675.1	324	43.5	7.6	18.5	5735.72	Makarieva et al. 2008

Supplementary Table S2. Assessing the substitution saturation using DAMBE for each of the 62 genes that failed to pass the filtering thresholds shown in the main text. Sequences are thought to have undergone little saturation when the index of substitution saturation (I_{ss}) is significantly ($P < 0.05$) smaller than the critical index of substitution saturation ($I_{ss.c}$) (see Methods in the main text). Significance code: ***, $P <$ 0.001. For each gene, the I_{ss} value is smaller than the corresponding $I_{ss.c}$ value (significant: 60 genes; non-significant: 2 genes). Therefore, following suggestions from Xia et al. (2003) and Oliveira et al. (2011), all genes are kept because of their usefulness (60 significant genes) or marginal usefulness (2 non-significant genes) for subsequent analysis.

**Family Id**	**Proportion of invariant sites**	I_{ss}	$I_{ss.c}$	***t*-value**	**Degree of freedom**	***P*-value (two-tailed)**
Cluster07945	0.0152	0.5494	2.2361	13.85	11	0***
Cluster08163	0.1037	0.4712	0.7851	21.79	1255	0***
Cluster08222	0.1903	0.3645	0.7968	28.22	1430	0***
Cluster08264	0.1857	0.484	0.77	15.06	869	0***
Cluster08314	0.2742	0.2998	0.7179	12.82	343	0***
Cluster08499	0.1615	0.4351	0.7577	14.74	726	0***
Cluster08578	0.1138	0.5263	0.7025	5.69	321	0***
Cluster08615	0.2189	0.4422	0.7359	12.45	479	0***
Cluster08635	0.1414	0.4118	0.7633	18.37	816	0***
Cluster08663	0.1662	0.324	0.7164	15.84	384	0***
Cluster08745	0.2566	0.2831	0.7391	16.72	479	0***
Cluster08828	0.5868	0.5014	0.7128	6.148	179	0***
Cluster08917	0.39	0.6391	0.7157	1.214	52	0.2301

Cluster09049	0.1231	0.4914	0.8006	21.65	1675	0***
Cluster09230	0.5763	0.5092	0.7277	6.455	230	0***
Cluster09296	0.0707	0.4661	1.0215	5.283	30	0***
Cluster09368	0.1847	0.3655	0.7567	19.37	694	0***
Cluster09396	0.3517	0.3814	0.721	12.08	322	0***
Cluster09428	0.1593	0.4261	0.79	22.26	1293	0***
Cluster09477	0.2332	0.4508	0.7684	16.29	795	0***
Cluster09521	0.195	0.3696	0.7636	18.82	769	0***
Cluster09562	0.197	0.3411	0.7753	23.75	941	0***
Cluster09606	0.2428	0.2561	0.6863	10.34	192	0***
Cluster09657	0.1424	0.4036	0.7665	19.22	861	0***
Cluster09739	0.1257	0.4318	0.6818	6.019	190	0***
Cluster09773	0.1935	0.3708	0.7716	20.71	885	0***
Cluster09985	0.2535	0.5461	0.7481	9.175	552	0***
Cluster10074	0.0716	0.4508	0.6818	6.369	202	0***
Cluster10089	0.132	0.2984	0.8082	40.69	1947	0***
Cluster10157	0.3298	0.4936	0.8235	31.73	2281	0***
Cluster10240	0.2402	0.4772	0.7248	9.054	398	0***
Cluster10384	0.5592	0.4616	0.6787	3.518	76	0.0007***
Cluster10681	0.1886	0.3006	0.7047	14.18	306	0***
Cluster11293	0.2274	0.3122	0.7617	21.67	715	0***
Cluster11427	0.223	0.3405	0.7132	11.92	339	0***

Cluster11465	0.1364	0.4284	0.7332	11.66	509	0***
Cluster11492	0.1762	0.3575	0.8119	38	2013	0***
Cluster11533	0.2989	0.5383	0.7498	10.01	533	0***
Cluster11543	0.1585	0.3931	0.764	19.14	809	0***
Cluster11626	0.114	0.3685	0.7265	12.65	475	0***
Cluster11627	0.5756	0.5407	0.7617	8.155	392	0***
Cluster11684	0.1572	0.4152	0.7695	18.61	892	0***
Cluster11798	0.2112	0.3153	0.7700	24.49	841	0***
Cluster12035	0.2534	0.3172	0.7156	11.58	339	0***
Cluster12064	0.279	0.5622	0.7667	10.76	726	0***
Cluster12067	0.1924	0.3375	0.8038	35.3	1649	0***
Cluster12073	0.125	0.2923	2.9752	16.36	7	0***
Cluster12425	0.4045	0.3816	0.7128	10.05	258	0***
Cluster12459	0.5749	0.4717	0.7468	9.004	308	0***
Cluster12578	0.1266	0.3045	0.7945	33.61	1472	0***
Cluster12733	0.1053	0.4737	0.8492	4.335	42	0.0001***
Cluster12822	0.1629	0.392	0.7894	24.79	1272	0***
Cluster12902	0.1904	0.3881	0.7281	11.55	443	0***
Cluster12935	0.1221	0.4467	0.7385	12.23	560	0***
Cluster12944	0.253	0.4288	0.7604	15.23	676	0***
Cluster12960	0.1443	0.6028	0.6834	1.536	112	0.1273
Cluster12963	0.2164	0.5526	0.7703	12.45	841	0***

Cluster13031	0.2295	0.6145	0.768	8.504	794	0***
Cluster13138	0.26	0.2968	0.7753	25.03	867	0***
Cluster13150	0.1959	0.3457	0.7388	15.49	515	0***
Cluster13426	0.2321	0.5083	0.7949	19.76	1303	0***
Cluster13434	0.1815	0.3553	0.7698	22.15	871	0***

Supplementary Table S3. Statistics of genomic features of the Chinese hook snout carp *Opsariichthys bidens*.

**S3a) Genome size**			
	**Genome survey**	**Initial assembly**	**Final assembly**
Base pairs	797 Mb	789 Mb	787 Mb
Contig N50 (bp)	263	6494884	6494884
Scaffold N50 (bp)	1866	6494884	22817007

**S3b) Function annotation of protein-coding genes**		
	**Number**	**Percent (%)**
Total	21375	100.00
Unannotated	431	2.02
Annotated	20944	97.98
by InterPro	19491	91.19
by GO	14842	69.44
by KEGG_ALL	20584	96.30
by KEGG_KO	12481	60.07
by Swissprot	18659	87.29
by TrEMBL	20827	97.44
by TF	3344	15.64
by Pfam	18860	88.23
by NR	20826	97.43

by KOG	17173	80.34

**S3c) Protein-coding gene size**	
	**Men length (bp)**
Gene	19745.53
CDS	1714.16
Exon	280.71
Intron	1734.22

**S3d) non-coding RNA size**				
	**Copy number**	**Mean length (bp)**	**Total length (bp)**	**Percent (%) of genome**
miRNA	487	84	41214	0.0052
tRNA	3677	75	277282	0.0352
rRNA	1519	129	196244	0.0249
snRNA	957	149	143428	0.0182

Supplementary Table S4. Pairwise comparison of mean value of molecular evolution rate (nucleotide substitution rate) of 5020 single-copy orthologous genes between one cypriniform fish (order Cypriniformes) and one non-cypriniform fish. *P*-value is corrected using the Tukey's HSD test. Species code for cypriniform fishes: Ccar represents *Cyprinus carpio*; Drer represents *Danio rerio*; Obid represents *Opsariichthys bidens*. Species code for non-cypriniform fishes: Ccha represents *Chanos chanos*; Char represents *Clupea harengus*; Espe represents *Etheostoma spectabile*; Gmor represents *Gadus morhua*; Omyk represents *Oncorhynchus mykiss*; Pnat represents *Pygocentrus nattereri*; Sacu represents *Syngnathus acus*; Smax represents *Scophthalmus maximus*; Xhel represents *Xiphophorus hellerii*. Significance code: **, adjusted $P < 0.01$; ***, adjusted $P < 0.001$. Substitution rate is expressed in the unit of substitution per site relative to root age (225.84 million years).

**Species1 (cypriniform fishes)**		**Species2 (non-cypriniform fishes)**		**Difference in substitution rate (Species1 – Species2)**	**adjusted *P*-value**
**Species code**	**Substitution rate**	**Species code**	**Substitution rate**		
Ccar	0.264	Ccha	0.128	0.136	0***
Ccar	0.264	Char	0.141	0.123	0***
Ccar	0.264	Espe	0.201	0.063	0***
Ccar	0.264	Gmor	0.164	0.100	0***
Ccar	0.264	Omyk	0.092	0.172	0***
Ccar	0.264	Pnat	0.168	0.096	0***
Ccar	0.264	Sacu	0.258	0.006	0.87
Ccar	0.264	Smax	0.173	0.091	0***
Ccar	0.264	Xhel	0.252	0.012	0.009**

Drer	0.280	Ccha	0.128	0.152	0***
Drer	0.280	Char	0.141	0.139	0***
Drer	0.280	Espe	0.201	0.079	0***
Drer	0.280	Gmor	0.164	0.116	0***
Drer	0.280	Omyk	0.092	0.188	0***
Drer	0.280	Pnat	0.168	0.112	0***
Drer	0.280	Sacu	0.258	0.022	0***
Drer	0.280	Smax	0.173	0.107	0***
Drer	0.280	Xhel	0.252	0.028	0***
Obid	0.290	Ccha	0.128	0.162	0***
Obid	0.290	Char	0.141	0.149	0***
Obid	0.290	Espe	0.201	0.089	0***
Obid	0.290	Gmor	0.164	0.126	0***
Obid	0.290	Omyk	0.092	0.198	0***
Obid	0.290	Pnat	0.168	0.122	0***
Obid	0.290	Sacu	0.258	0.032	0***
Obid	0.290	Smax	0.173	0.117	0***
Obid	0.290	Xhel	0.252	0.038	0***

References

Barrionuevo WR, Burggren WW. 1999. O_2 consumption and heart rate in developing zebrafish (*Danio rerio*): influence of temperature and ambient O_2. *Am J Physiol Regul Integr Comp Physiol*, **276**(2): R505-R513.

Brown JAG, Jones A, Matty AJ. 1984. Oxygen metabolism of farmed turbot (*Scophthalmus maximus*): I. the influence of fish size and water temperature on metabolic rate. *Aquaculture*, **36**(3): 273-281.

Duncan WP. 2020. Interspecific differences in the metabolic rate, gill dimension and hematology of fish in an Amazonian Floodplain Lake. *Aquat Sci Technol*, **8**(1): 38-58.

Fu SJ, Peng Z, Cao ZD, et al. 2012. Habitat-specific locomotor variation among Chinese hook snout carp (*Opsariichthys bidens*) along a river. *PLoS One*, **7**(7): e40791.

Johnstone ADF, Wardle CS, Almatar SM. 1993. Routine respiration rates of Atlantic mackerel, *Scomber scombrus* L., and herring, *Clupea harengus* L., at low activity levels. *J Fish Biol*, **42**(1): 149-151.

Makarieva AM, Gorshkov VG, Li BL, et al. 2008. Mean mass-specific metabolic rates are strikingly similar across life's major domains: evidence for life's metabolic optimum. *Proc Natl Acad Sci USA*, **105**(44): 16994-16999.

Oliveira C, Avelino GS, Abe KT, et al. 2011. Phylogenetic relationships within the speciose family Characidae (Teleostei: Ostariophysi: Characiformes) based on multilocus analysis and extensive ingroup sampling. *BMC Evol Biol*, **11**: 275.

Xia X, Xie Z, Salemi M, et al. 2003. An index of substitution saturation and its application. *Mol Phylogenet Evol*, **26**(1): 1-7.
